# Identification of distinct human invariant natural killer T-cell response phenotypes to alpha-galactosylceramide

**DOI:** 10.1186/1471-2172-9-71

**Published:** 2008-12-03

**Authors:** Joanne E Croudace, Stuart M Curbishley, Manuela Mura, Carrie R Willcox, Petr A Illarionov, Gurdyal S Besra, David H Adams, David A Lammas

**Affiliations:** 1 MRC Centre for Immune Regulation, University of Birmingham, Birmingham B15 2TT, UK; 2 School of Biosciences, University of Birmingham, Birmingham, B15 2TT, UK

## Abstract

**Background:**

Human CD1d-restricted, invariant natural killer T cells (iNKT) are a unique class of T lymphocytes that recognise glycolipid antigens such as α-galactosylceramide (αGalCer) and upon T cell receptor (TCR) activation produce both Th1 and Th2 cytokines. iNKT cells expand when cultured *in-vitro *with αGalCer and interleukin 2 (IL-2) in a CD1d-restricted manner. However, the expansion ratio of human iNKT cells varies between individuals and this has implications for attempts to manipulate this pathway therapeutically. We have studied a panel of twenty five healthy human donors to assess the variability in their *in-vitro *iNKT cell expansion responses to stimulation with CD1d ligands and investigated some of the factors that may influence this phenomenon.

**Results:**

Although all donors had comparable numbers of circulating iNKT cells their growth rates *in-vitro *over 14 days in response to a range of CD1d ligands and IL-2 were highly donor-dependent. Two reproducible donor response patterns of iNKT expansion were seen which we have called 'strong' or 'poor' iNKT responders. Donor response phenotype did not correlate with age, gender, frequency of circulating iNKT, or with the CD1d ligand utilised. Addition of exogenous recombinant human interleukin 4 (IL-4) to 'poor' responder donor cultures significantly increased their iNKT proliferative capacity, but not to levels equivalent to that of 'strong' responder donors. However in 'strong' responder donors, addition of IL-4 to their cultures did not significantly alter the frequency of iNKT cells in the expanded CD3^+ ^population.

**Conclusion:**

(i) *in-vitro *expansion of human iNKT cells in response to CD1d ligand activation is highly donor variable, (ii) two reproducible patterns of donor iNKT expansion were observed, which could be classified into 'strong' and 'poor' responder phenotypes, (iii) donor iNKT response phenotypes did not correlate with age, gender, frequency of circulating iNKT cells, or with the CD1d ligand utilised, (iv) addition of IL-4 to 'poor' but not 'strong' responder donor cultures significantly increased their *in-vitro *iNKT cell expansion to αGalCer.

## Background

iNKT cells are a minor subpopulation of the human peripheral blood T cell repertoire. They are members of the innate lymphocyte subset, which includes natural killer cells (NK) and γ/δT cells. Both mouse and human iNKT cells, unlike non-variant NKT, express an invariant TCR and recognise lipid antigens in association with the HLA class Ib molecule CD1d. Human iNKT cells express a combination of Vα24Vβ11 [1] and in mouse Vα14Vβ2,7 or 8 [2]. The precise physiological role of iNKT cells is uncertain but they are involved in the activation of dendritic cells (DC) in response to microbial lipid antigens and thereby provide a link between innate and adaptive immune responses to infection [3-5]. They are also thought to recognise self-lipid antigens presented on CD1d and hence to play a role in tolerance induction and the suppression of autoimmunity [6-8]. Conversely, iNKT cells are involved in cancer immunosurveillance, recognising altered self lipid antigens expressed by malignant cells and eliminating them at an early stage of transformation [9].

iNKT cells are universally responsive to αGalCer, a glycosphingolipid antigen derived from the marine sponge *Agelas mauritanius*, or to its synthetic analogue KRN7000 [10]. αGalCer binds to the hydrophobic groove of CD1d and activates all iNKT cells by means of TCR recognition [9]. Responding cells proliferate and characteristically release both Th1 and Th2 cytokines.

αGalCer has been reported to promote DC maturation and subsequent antigen-specific T cell responses via selective engagement of iNKT cells. DC maturation is then enhanced via CD40L expression by activated iNKT cells and via their release of IFNγ resulting in IL-12 production and HLA upregulation by DC in a positive feed back manner with consequent promotion of antigen presentation [9].

This adjuvant effect is thought to underlie the observations made in a number of mouse-models that αGalCer promotes specific anti-tumour immunity and clearance of established tumours [11-13]. In turn this has led to the proposed use of iNKT cell ligands like αGalCer as adjuvants for human tumour immunotherapy [12,13].

However, iNKT cells are reduced in both number and activity in patients with certain malignancies [14-19]. Moreover, *in-vivo*, vaccination with soluble αGalCer leads to only transient activation of iNKT cells followed by long-term unresponsiveness [20]. Thus the optimal use of αGalCer-based immunotherapy for cancer patients is envisaged to involve infusing αGalCer *in-vitro*-expanded autologous iNKT cells followed by αGalCer-pulsed, tumour antigen-loaded DCs.

Quantitative defects in iNKT cells are predictive of progression in certain autoimmune diseases. For example, iNKT cells are reduced in diabetes-prone NOD mice and increasing iNKT cell numbers by adoptive transfer [21] or via the introduction of a *Vα14-Jβ18 *transgene suppresses subsequent disease progression [22].

All iNKT cells expand when cultured *in-vitro *with αGalCer and IL-2 in a CD1d-restricted manner [23] and *in-vivo *following administration of αGalCer-pulsed DCs [24]. However, the expansion ratio of human iNKT cells is known to be highly variable between individuals [25-27].

In this study we have evaluated the iNKT expansion profiles of a panel of twenty five healthy human donors to assess the degree of individual variability on stimulation with various CD1d ligands. We also sought to define some of the factors that may influence such donor variation.

## Methods

### Patient samples

All donor blood samples were obtained from Queen Elizabeth Hospital, Edgbaston, Birmingham UK, following patient consent and local Ethics Approval.

### Antibodies and flow cytometric analysis

Human iNKT cells were identified using a novel monoclonal antibody (clone 6B11) specific for the CDR3 loop of the human Vα24Jα18 TCR alpha chain which has been previously reported to specifically identify iNKT cells [28,29] referred to subsequently as 6B11. Other antibodies comprise CD3-APC, CD4-PerCP and CD8α-FITC (BD Biosciences). For surface staining, cells were washed with staining buffer (PBS + 2% FCS), and incubated for 30 mins, on ice in the dark with the relevant antibody combinations. Cells were then washed twice with staining buffer and analysed by flow cytometry (BD Coulter). Data was collected using a FACS Calibur (BD Biosciences) and analysed by "Flowjo" flow cytometry software (Tree Star, Inc).

### Glycolipids

αGalCer (C26:0) and PI-3 (C20:2) were synthesised within GSB's laboratory by PAI, as previously described [30]. The glycolipids were dissolved in DMSO at a concentration of 100 μg/ml, and diluted into culture medium to the required final concentration.

### iNKT cell proliferation assays to CD1d ligands

Peripheral blood mononuclear cells (PBMC) were isolated from buffy coats by Ficoll density gradient centrifugation. Cells were cultured in RPMI 1640 + L-glutamine (2 mM) + 10% human serum (Lonza) + recombinant human IL-2 (IL-2) 100 u/ml (Peprotec) in the presence of αGalCer (C26:0) at a final concentration of either: 50, 100 or 500 ng/ml or with PI-3 (C20:2) at 100 ng/ml, for a 14-day culture period. Cell cultures were fed by replacement of half volume media twice weekly with fresh RPMI supplemented with 1% penicillin and streptomycin, 10% human serum and IL-2 (200 u/ml). No additional glycolipid was added during feeding of cells. The percentage of iNKT cells within donor populations were assessed by flow cytometry performed on days 0 (to allow comparison of peripheral iNKT frequency), 7, 10 and 14 days post-culture. Co-expression of 6B11 (an antibody specific to the Vα24Jα18 iNKT TCR) and CD3 were used to identify the iNKT population in each culture [31]. For confirmation of iNKT proliferation, PBMC were stained with CFSE dissolved in DMSO for 10 mins prior to incubation with αGalCer/IL-2 and proliferation of iNKT was analysed by dilution of the CFSE signal/6B11^+ ^staining on day 14 post culture. For analysis of subpopulations of iNKT, cells were surface stained with 6B11-PE, CD3-APC, CD4-PerCP and CD8α-FITC.

### Analysis of effects of age, gender, and peripheral iNKT cell frequency

To assess whether donor gender and/or age affected iNKT expansion, PBMC were isolated from donor blood samples and iNKT cells allowed to expand in culture with αGalCer/IL-2 over 14 days for flow cytometric assessment, as previously described. Peripheral iNKT levels were assessed by analysing the number of 6B11^+ ^cells within the CD3^+ ^population of the PBMC of each donor examined in the study on day 0.

### Effects of adding exogenous IL-4 to 'poor' and 'strong' responder' iNKT cultures

PBMC were cultured with αGalCer and IL-2 (100 u/ml), from the blood of identified 'poor' and 'strong' responder donors as previously described. Exogenous recombinant human IL-4 (IL-4) (10 ng/ml, Peprotec) was added to the PBMC cultures at day 0 and the percentage of iNKT cells present at day 14 post-culture assessed by flow cytometry. IL-4 was added throughout the 14 day culture period being added to fresh media (20 ng/ml) prior to feeding of cells, as previously described.

### Statistical Analysis

Data was tested for normal distribution (Shapiro Wilk). For data with normal distribution Students T tests were carried out (Excel spreadsheets). For data with non-normal distribution a non-parametric Man Whitney U test (SPSS) was performed, which does not assume normal distribution. P-values < 0.05 were considered as significant (95% confidence). All error bars represent the standard error of the mean.

## Results and discussion

### iNKT cell proliferative responses to αGalCer are donor-dependent

**Figure 1:**
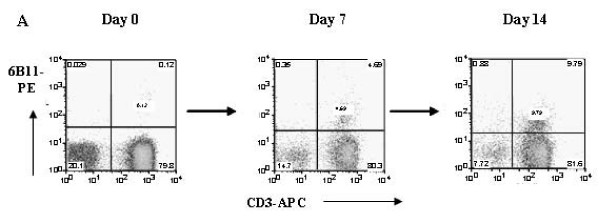
**Identification of two distinct donor-dependent, iNKT expansion phenotypes**. This figure illustrates figure 1A only, see additional file 1 for full figure including figures 1A, B, C and D. (A) Representative FACS plots illustrating expansion of PBMC-derived iNKT cells (CD3^+^/6B11^+^) in response to αGalCer + IL-2 over 7 and 14 days.

Stimulation of PBMC with αGalCer + IL-2 resulted in expansion of the starting iNKT population over a 14-day culture period (Fig 1A and see additional file 1). However, the magnitude of the response was highly variable between donors with some cultures exhibiting only a minimal increase in the percentage of iNKT cells above their starting levels (Fig 1B, see additional file 1). Overall, a marked dichotomy in the ability of peripheral blood-derived iNKT cells to expand *in-vitro *to αGalCer and IL-2 was observed (Fig 1B, see additional file 1). Subjects could be divided into those whose iNKT cells expanded significantly over a 14-day culture period (from approx 0.2% iNKT to 4–18% of the CD3^+ ^population), or those in whom no or minimal expansion was observed (i.e. from 0.2 to < 2% of the CD3^+ ^population) (Fig 1B, see additional file 1, n = 25). The expansion rate of the former being equivalent to a 40–514 fold increase from the starting population while in the latter this equated only to an expansion rate of < 20 fold. iNKT cells were identified using a novel monoclonal antibody (clone 6B11) specific for the CDR3 loop of the human Vα24Jα18 TCR alpha chain, which has previously been reported to selectively label iNKT cells [28,29].

The increase in the percentage of iNKT cells within 'strong' responder cell cultures over 14 days was shown by 5(6)-carboxyfluorescein diacetate N-succinimidyl ester (CFSE) staining to result from proliferation of the iNKT cells (Fig 1C, see additional file 1) rather than to their selective survival within the total CD3^+ ^population. Similarly the lack of increase in the percentage of iNKT cells within 'poor' responders was associated with an absence of proliferation (Fig 1C, see additional file 1). However iNKT cells were still identifiable within 'poor' responder cultures on co-staining with 6B11^+ ^and CD3^+ ^following 14 days of expansion. They were also CFSE positive indicating that initial peripheral iNKT cells were still present within 'poor' responder cultures but had not divided as well as those in 'strong' responder cultures in response to αGalCer.

The type of response mounted by a given donor to αGalCer was also stable and highly reproducible over time when the same subjects were assessed on multiple occasions (Fig 1D, see additional file 1). The data presented are representative of 2 individuals – 1 'strong' responder and 1 'poor' responder donor. To date, five different donors have been assayed on three separate occasions, and have exhibited a similar consistency in their response profiles on each occasion.

The results may have clinical implications as several phase I clinical studies have been carried out in cancer patients using intravenous αGalCer or αGalCer-loaded DCs, with limited success [32,17,20,33]. This has been attributed to a number of different factors including: inefficient delivery of αGalCer or αGalCer-loaded DCs to the tumour site, the low numbers of peripheral iNKT cells found in cancer patients, or to the effects of various pre-treatment drugs on iNKT and/or their CD1d antigen-presenting cells [18]. However, our results suggest that the donor's inherent iNKT response phenotype may also affect their clinical response. By understanding how iNKT cell expansion can be manipulated in both response phenotypes it may prove possible to greatly improve the effectiveness of αGalCer as an immunotherapeutic adjuvant. Moreover by understanding what factors regulate iNKT cell growth *in-vitro *it may prove possible to manipulate their expansion in 'poor' responder cancer patients to improve the effectiveness of using autologous iNKT cell infusion as an adjunct to DC immunotherapy.

### Analysis of iNKT subpopulations in response to αGalCer

**Figure 2:**
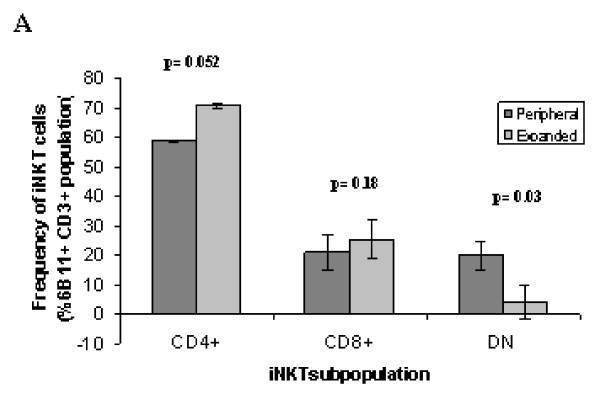
**iNKT cell proliferative responses – Effects of iNKT cell subsets, different CD1d ligands and aGalCer concentration**. This figure illustrates figure 2A only, see additional file 2 for full figure including figures 2A, B, and C. (A) Percentage of iNKT subpopulations in the total peripheral iNKT cell population and expanded iNKT cell population after 14 days in culture with αGalCer + IL-2 (n = 3 +/- standard error of the mean).

The relative percentages of the three known iNKT cell subpopulations (i.e. CD4^+^, CD8^+ ^and CD4^-^CD8^- ^(double negative, DN)) were analysed before and after expansion with αGalCer (Fig 2A and see additional file 2). The study was undertaken to determine whether the *in-vitro *conditions used to expand the iNKT cells altered or promoted the proliferation of any particular iNKT subset. After 14 days in culture, all 3 subsets were still identifiable in the resultant population. However the expanded CD4^+ ^subset represented a higher proportion of the total iNKT population (70%) as compared with their peripheral levels (approximately 60% of the total iNKT population). In contrast the expanded DN iNKT subset represented a lower proportion of the total iNKT population (<5%) as compared with their peripheral levels (approximately 20% of the total iNKT population) (Fig 2A and see additional file 2). Hence CD4^+ ^cells were the predominant phenotype in both the starting and expanded iNKT cell populations. This is consistent with the findings of Lin and colleagues who observed that in the presence of IL-2 the majority of iNKT cells that responded to αGalCer were CD4^+ ^[34]. Thus following stimulation of donor PBMC with a combination of αGalCer + IL-2 all three subpopulations of iNKT cells were still present and identifiable.

### iNKT cells expand to different CD1d ligands

Interest in the potential to manipulate iNKT cells for therapeutic purposes has been aroused since the demonstration that αGalCer could induce iNKT cell activation, proliferation and secretion of Th1 and Th2 cytokines. The development of multiple glycolipid analogues with different capacities to stimulate iNKT cells has also increased their potential therapeutic utility [30,35]. We therefore studied the kinetics of iNKT cell proliferative responses in PBMC cultures derived from 'strong' and 'poor' responsive donors to both αGalCer (C26.0) and to its analogue PI-3 (C20:2) (Fig 2B, see additional file 2). The results show that for each donor the kinetics of their iNKT cell proliferative response was similar to both analogues (Fig 2B, see additional file 2, n = 6). The expansion kinetics of two 'strong' responders (Fig 2Bi &2Bii, see additional file 2) and two 'poor' responders (Fig 2Biii &2Biv, see additional file 2) are illustrated. The fact that the same donor-dependent response phenotype was observed with another CD1d ligand PI-3, which contains a shorter, diunsaturated fatty acid chain [30,8], suggests that iNKT cell proliferation is pre-programmed and not ligand-dependent. PI-3 was used because it is a potent human iNKT cell antigen, which, unlike αGalCer promotes Th2-biased rather than Th1 responses *in-vitro *and *in-vivo *in mice [7,9]. Thus, although different CD1d ligands have been reported to influence the cytokine profiles of iNKT cells [6,7] we did not observe any related effect on iNKT cell expansion efficiency. The findings suggest that donor-dependent factors are more critical than specific glycolipid/CD1d/TCR interactions in determining the degree of *in-vitro *iNKT cell proliferative responses.

### Effects of αGalCer concentration on iNKT cell expansion

A titration of αGalCer was performed to determine whether the concentration of the CD1d ligand affected subsequent iNKT cell proliferation. iNKT expansion assays were set up to determine the effects of different concentrations of αGalCer (i.e. 500, 100 and 10 ng/ml), using cells derived from previously defined 'strong' responder donors (Fig 2C, see additional file 2, n = 3). 100 ng/ml of αGalCer, the dose cited in the literature for most iNKT cell functional studies, elicited the maximum iNKT proliferative response [9] (Fig 2C, see additional file 2). Hence 100 ng/ml was used for all further iNKT cell expansion studies. However, titration of the dose of αGalCer against cells derived from a 'poor' responder donor did not unmask a proliferative response at either lower or higher αGalCer concentrations (results not shown) suggesting that the iNKT cell response phenotypes identified in this study were not dependent on the concentration of the CD1d ligand used.

### Assessment of peripheral levels of iNKT, donor age and gender on iNKT cell expansion phenotype

**Figure 3:**
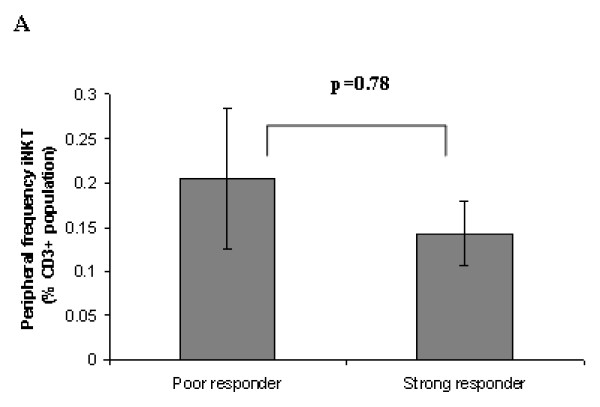
**Donor iNKT response phenotypes are not associated with peripheral iNKT levels, donor age or gender**. This figure illustrates figure 3A only, see additional file 3 for full figure including figures 3A, B, C and D. (A) Comparison of peripheral iNKT cell levels between 'strong' and 'poor' iNKT responsive donor groups (n = 15 'poor' and n = 10 for 'strong' +/- standard error of the mean).

The relationship, between the proportions of iNKT cells in donor peripheral blood and the functional capacity of the iNKT's to expand has not been previously determined. This lead us to investigate whether differences in the starting frequency of iNKT cells within the initial non-adherent CD3^+ ^lymphocyte population of both 'strong' and 'poor' responder donors might explain their different expansion responses. Analysis of human iNKT cells is particularly demanding since their frequency among peripheral blood T cells is relatively low ranging from < 0.01–1.1% of the total CD3+ population, with a mean of approximately 0.2% [36]. No discernable differences were observed in the percentage of the starting iNKT cell populations between the different responder donor phenotypes (Fig 3A and see additional file 3), with 'strong' responder peripheral frequencies ranging from 0.02–0.3% (average 0.14%, n = 10) and 'poor' responder frequencies from 0.05–1.07% (average 0.2%, n = 15), indicating that the initial numbers of iNKT cells in the blood does not determine subsequent iNKT cell expansion efficiency (Fig 3A and see additional file 3). In support of this finding we observed both poor expansion of iNKT cells in donors with relatively high peripheral levels of iNKT cells and conversely strong expansion of iNKT cells in donors with relatively low peripheral levels of iNKT cells (Fig 3B, see additional file 3). These findings are consistent with Crough and colleagues who also failed to observe any relationship between the iNKT cell percentage in healthy donors and the degree to which their iNKT cells expanded in culture to αGalCer [26]. However, it is thought that the low number of circulating iNKT cells in cancer patients does contribute to their lack of immune reactivity to αGalCer vaccination [17] as immune activation was observed in only those patients who had relatively normal pre-treatment, peripheral iNKT cell numbers [17].

Previous studies have suggested that differences in iNKT cell proliferation efficiency are associated with the age of the donor [37,38,26] reflecting presumably either a reduction in their proliferative capacity, clonal exhaustion or reduced thymic output. Consistent with these latter reports a discernable decline in the magnitude of the iNKT cell proliferative response with age with a reduction in the frequency of iNKT (day 14) was observed in older donors (Fig 3C, see additional file 3). However no significant correlate between the two was identified (n = 20, R^2 ^= 0.11) and both 'strong' and 'poor' response phenotypes were still discernable in both young and elderly donors (Fig 3C, see additional file 3).

As there have been no previous reports on whether gender effects iNKT cell expansion the response phenotypes of a cohort of eleven male and eight female cell donors were assessed (Fig 3D, see additional file 3). Both 'strong' and 'poor' iNKT responders were identified within each gender group (Fig 3D, see additional file 3) with average iNKT cell frequencies of 4.78% and 3.45% (P = 0.71) respectively by day 14, suggesting no major bias between males and females in their iNKT response phenotypes to αGalCer. However, there was an observed trend towards greater numbers of low responders amongst female donors, which merits further investigation on larger donor cohorts.

### IL-4 promotes iNKT cell expansion in 'poor' but not in 'strong' responders

**Figure 4:**
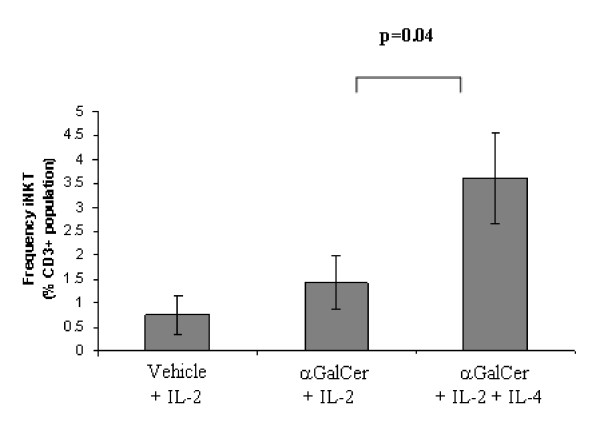
**Effects of exogenous IL-4 on expansion of iNKT cells in 'poor' and 'strong' responder donors**. This figure illustrates figure 4A only, see additional file 4 for full figure including figures 4A and B. (A) Levels of PBMC-derived iNKT cells from 'poor' responder donors after 14-days of culture in the presence of: (i) vehicle (DMSO) + IL-2, (ii) αGalCer + IL-2, or (iii) αGalCer + IL-2 + IL-4 (n = 4, +/- standard error of the mean).

A recent study in mice has reported that exogenous IL-4 augments iNKT cell proliferation early in the culture period of poor responsive strains whereas IFNγ inhibited iNKT cell proliferation suggesting that it is the ratio of IL-4 and IFNγ, which is important in defining iNKT cell expansion [39]. Thus we investigated whether addition of exogenous IL-4 could induce stronger iNKT cell proliferative responses in PBMC cultures derived from 'poor' responder donors. Addition of recombinant human IL-4 (10 ng/ml) to 'poor' responder donor cultures increased iNKT cell expansion from approximately 1.4% to 3.6% of the total CD3^+ ^population over 14 days (n = 4, p = 0.04) (Fig 4A and see additional file 4). Thus IL-4 contributes to the donor-dependent heterogeneity observed in iNKT expansion to αGalCer. Interestingly however addition of IL-4 (10 ng/ml) to 'strong' responder donor PBMC cultures did not promote iNKT cell proliferation. In fact, IL-4 appeared to reduce the frequency of iNKT cells in the CD3^+ ^population following 14 days culture, but this affect was not significant (Fig 4B, see additional file 4).

The ability of IL-4 to promote iNKT cell expansion in 'poor' responder cultures may reflect the fact that GATA-3 has been reported to be critical for their peripheral development, function and survival [40] although iNKT maturation is reportedly controlled by the T_H_1 transcription factor T-bet [41]. In addition, immature neonatal iNKT cells have a greater ability to proliferate compared with more mature iNKT cells [37,42]. In mice, cytokine analysis of the developmental stages of iNKT cells have revealed a T_H_2 to T_H_1 conversion, suggesting that the functions of iNKT cells may be developmentally controlled [42]. Therefore 'poor' responder donors may prove to have fewer circulating immature iNKT cells than 'strong' responder donors. However, if exogenous IL-4 selectively expands immature iNKT cells, the lack of expansion of iNKT in 'poor' responders to αGalCer may be attributable to a greater number of non-responsive immature cells in such individuals. Support for this comes from the fact that adult, as opposed to neonatal iNKT cells, are oligoclonally expanded [43], which results in increased numbers of iNKT cells after birth in mice [44] and higher numbers of iNKT cells in adult blood compared with cord blood in humans [37]. The latter reports suggest that mature iNKT cells continue to expand after birth presumably in response to stimulation by self- and/or environmental antigens presented on CD1d and exist as chronically activated cells. Whether IL-4 and/or IFNγ production or the overall balance of these two opposing cytokines is responsible for the inter-individual differences observed in this study remains to be determined.

## Conclusion

We detected marked but reproducible donor-dependent differences in the expansion of blood iNKT cells in response to *in-vitro *culture with αGalCer and IL-2. Individual donors could be classified either as 'strong' or 'poor' responders in terms of their relative iNKT expansion efficiencies. The differences in the two response phenotypes could not be explained by peripheral levels of iNKT, the age or sex of the donor, or to the type or concentration of the CD1d ligand used. However, reduced iNKT proliferative responses were observed in older donors but not to a significant degree. Expansion *in-vitro *of iNKT cells derived from 'poor' responder donors was augmented by addition of exogenous IL-4 to the cell cultures. The results suggest that individual donor iNKT cell response phenotypes may be associated with inherent early differential production of IL-4 and/or other Th2 cytokines within the cell cultures.

The inherent differences observed in iNKT cell responses between individuals, could potentially influence their susceptibility to diseases in which iNKT cells are implicated, including autoimmune diseases, malignancies and infections. The results also suggest that the efficacy of immunotherapy with αGalCer/DC-based vaccines may depend upon the recipients' inherent iNKT cell response phenotype. Reconstitution of cancer patients exhibiting a 'poor' iNKT response phenotype with cytokine-induced, *in-vitro *expanded, autologous iNKT cells may then help to boost their subsequent response to immunotherapy with αGalCer/DC-based vaccines.

## Abbreviations

iNKT: invariant natural killer T cell; αGalCer: alpha-galactosylceramide; DC: Dendritic cell; PI-3: C20:2 analogue of alpha-galactosylceramide; PBL: peripheral blood lymphocytes; PBMC: peripheral blood mononuclear cells.

## Authors' contributions

JEC carried out all the experiments. αGalCer and PI-3 were synthesised by PAI in GSB laboratory. Experimental design and manuscript preparation was performed by: JEC, MM, SMC, CRW, DAL and DHA. All authors have read and approved the manuscript.

## Supplementary Material

Additional file 1 **Identification of two distinct donor-dependent, iNKT expansion phenotypes**. (A) Representative FACS plots illustrating expansion of PBMC-derived iNKT cells (CD3^+^/6B11^+^) in response to αGalCer + IL-2 over 7 and 14 days. (B) Donor response profiles illustrating two distinct iNKT cell proliferation phenotypes to αGalCer + IL-2 over 14 days (n = 25). (C) Representative CFSE dilution profiles of PBMC-derived iNKT cells from a 'strong' and a 'poor' αGalCer responder donor (n = 6, for each phenotype), (D) Reproducibility of individual donor iNKT response phenotype to αGalCer expansion over 14 days from PBMC – illustration of iNKT cell responses obtained in one 'strong' and one 'poor' responder donor, each tested on 3 separate occasions. Click here for file

Additional file 2 **iNKT cell proliferative responses – Effects of iNKT cell subsets, different CD1d ligands and aGalCer concentration**. (A) Percentage of iNKT subpopulations in the total peripheral iNKT cell population and expanded iNKT cell population after 14 days in culture with αGalCer + IL-2 (n = 3 +/- standard error of the mean), (B) Comparison of iNKT cell proliferation to αGalCer + IL-2 and a structural analogue PI-3 (C20:2) (αGalCer = ■, PI-3 = ▲, DMSO Vehicle = ◆) over 14 days in 2 'strong' (i&ii) and 2 'poor' responder donors (iii & iv) (n = 6) (C) iNKT cell expansion kinetics to 3 concentrations of αGalCer + IL-2 (ie 500 ng/ml = ▲, 100 ng/ml = ■, 50 ng/ml = ◆) in three 'strong' responder donors over 14 days (n = 3). Click here for file

Additional file 3 **Donor iNKT response phenotypes are not associated with peripheral iNKT levels, donor age or gender**. (A) Comparison of peripheral iNKT cell levels between 'strong' and 'poor' iNKT responsive donor groups (n = 15 'poor' and n = 10 for 'strong' +/- standard error of the mean), (B) FACS plots illustrating relatively low peripheral levels in a 'strong' responder donor and relatively high peripheral levels in a 'poor' responder donor, (C) Comparison of donor age with iNKT cell expansion efficiency (i.e. % iNKT in total CD3^+ ^population following 14 days of culture with αGalCer + IL-2), (n = 20) (D) Percentage of iNKT cells induced in 14-day PBMC cultures of 11 male and 8 female donors in response to αGalCer/IL-2. Click here for file

Additional file 4 **Effects of exogenous IL-4 on expansion of iNKT cells in 'poor' and 'strong' responder donors**. (A) Levels of PBMC-derived iNKT cells from 'poor' responder donors after 14-days of culture in the presence of: (i) vehicle (DMSO) + IL-2, (ii) αGalCer + IL-2, or (iii) αGalCer + IL-2 + IL-4 (n = 4, +/- standard error of the mean), (B) Levels of PBMC-derived iNKT cells from 'strong' responder donors after 14-days of culture in the presence of: (i) vehicle (DMSO) + IL-2, (ii) αGalCer + IL-2, or (iii) αGalCer + IL-2 + IL-4 (n = 3, +/- standard error of the mean). Click here for file
