# Supplementary figures and images for: Identification of distinct human invariant natural killer T-cell response phenotypes to alpha-galactosylceramide
Source: BMC Immunol. 2008 Dec 3;9:71. doi: 10.1186/1471-2172-9-71 (PMC2613383; doi:10.1186/1471-2172-9-71)

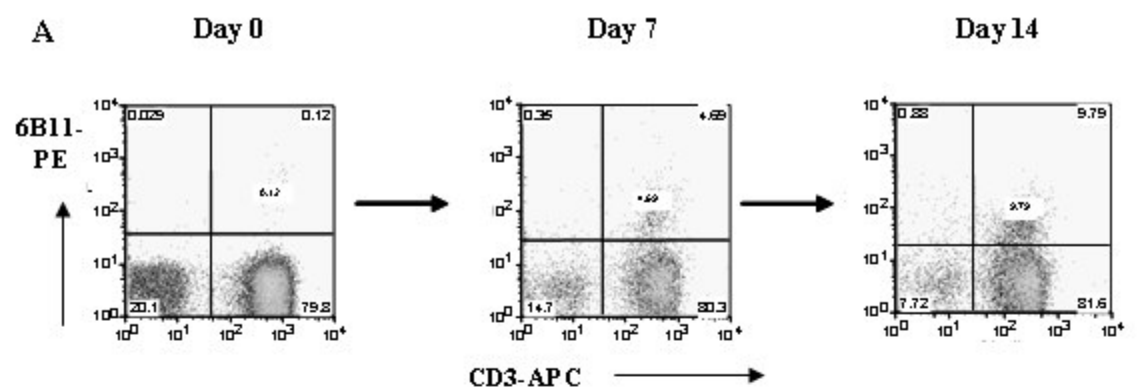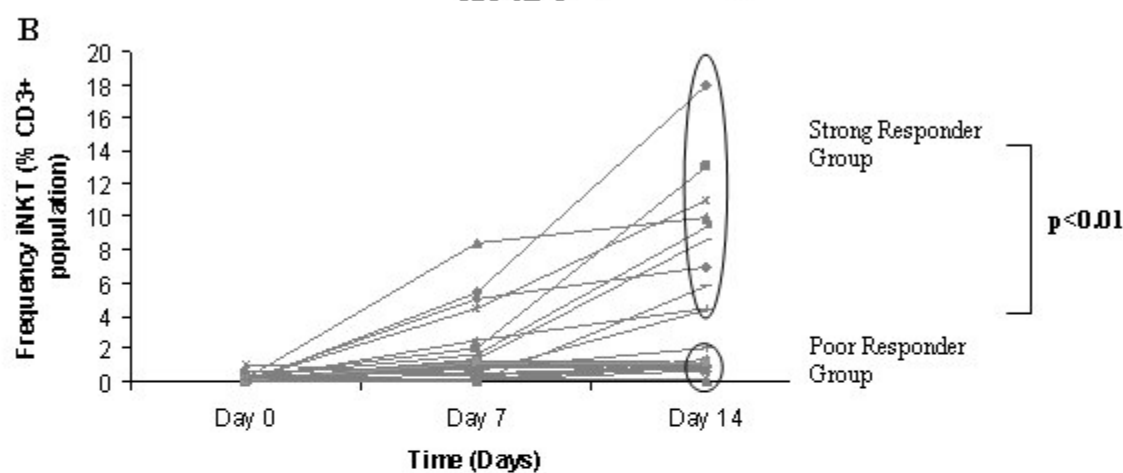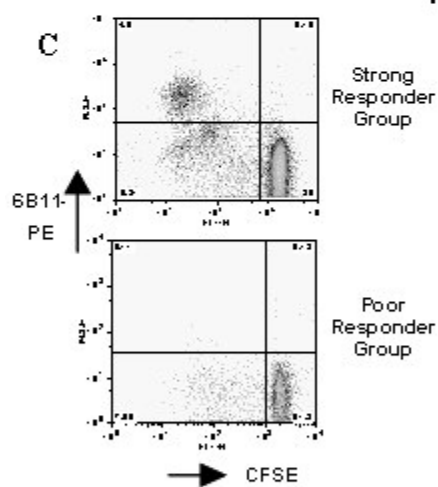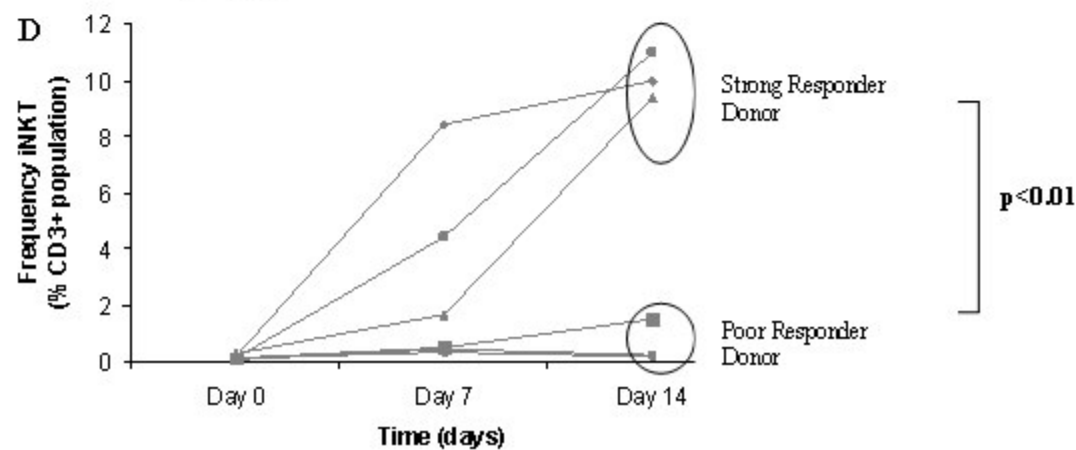

Supplement: Additional file 1 — Identification of two distinct donor-dependent, iNKT expansion phenotypes. (A) Representative FACS plots illustrating expansion of PBMC-derived iNKT cells (CD3+/6B11+) in response to αGalCer + IL-2 over 7 and 14 days. (B) Donor response profiles illustrating two distinct iNKT cell proliferation phenotypes to αGalCer + IL-2 over 14 days (n = 25). (C) Representative CFSE dilution profiles of PBMC-derived iNKT cells from a 'strong' and a 'poor' αGalCer responder donor (n = 6, for each phenotype), (D) Reproducibility of individual donor iNKT response phenotype to αGalCer expansion over 14 days from PBMC – illustration of iNKT cell responses obtained in one 'strong' and one 'poor' responder donor, each tested on 3 separate occasions. [file 1471-2172-9-71-S1.pdf]

**A**

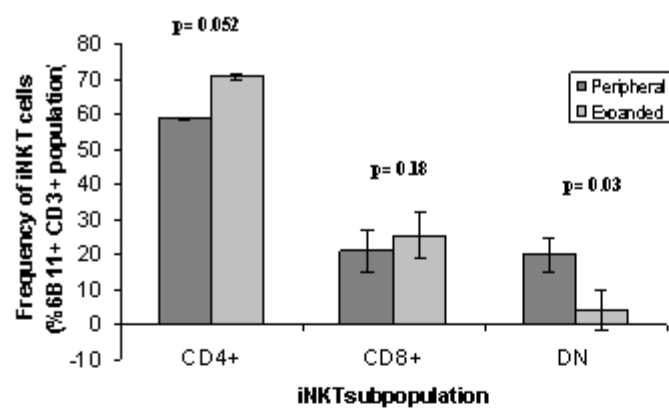

**B**

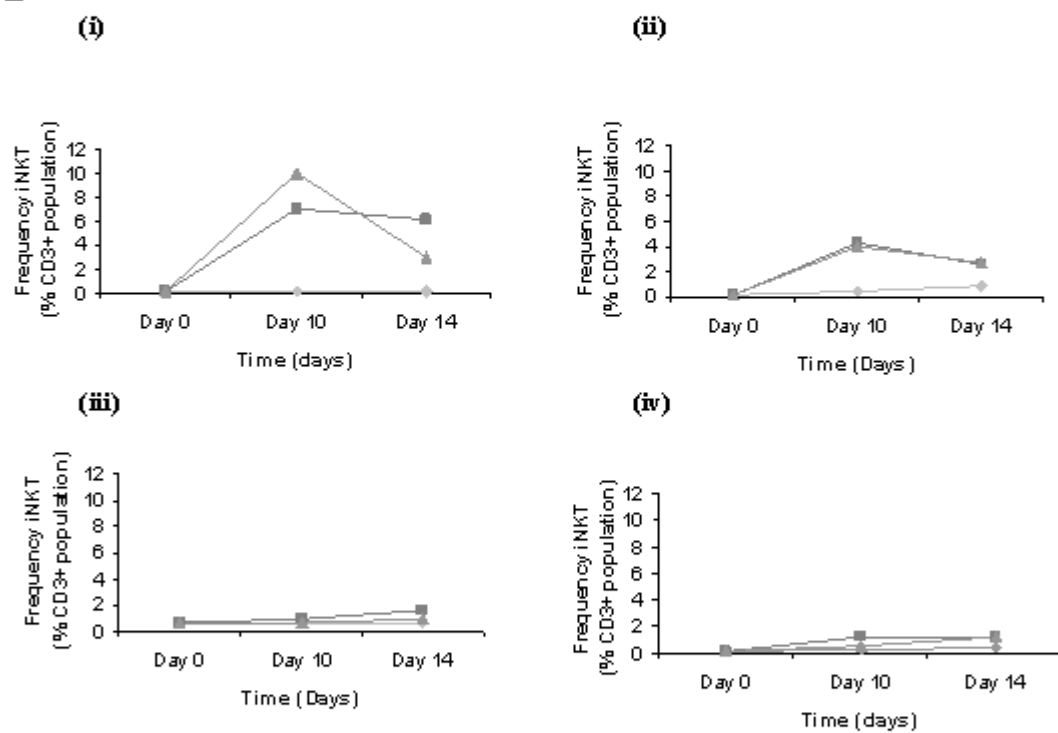

**C**

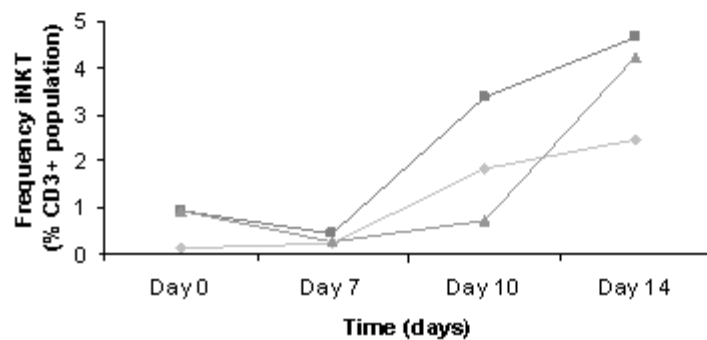

Supplement: Additional file 2 — iNKT cell proliferative responses – Effects of iNKT cell subsets, different CD1d ligands and aGalCer concentration. (A) Percentage of iNKT subpopulations in the total peripheral iNKT cell population and expanded iNKT cell population after 14 days in culture with αGalCer + IL-2 (n = 3 +/- standard error of the mean), (B) Comparison of iNKT cell proliferation to αGalCer + IL-2 and a structural analogue PI-3 (C20:2) (αGalCer = ■, PI-3 = ▲, DMSO Vehicle = ◆) over 14 days in 2 'strong' (i&ii) and 2 'poor' responder donors (iii & iv) (n = 6) (C) iNKT cell expansion kinetics to 3 concentrations of αGalCer + IL-2 (ie 500 ng/ml = ▲, 100 ng/ml = ■, 50 ng/ml = ◆) in three 'strong' responder donors over 14 days (n = 3). [file 1471-2172-9-71-S2.pdf]

A

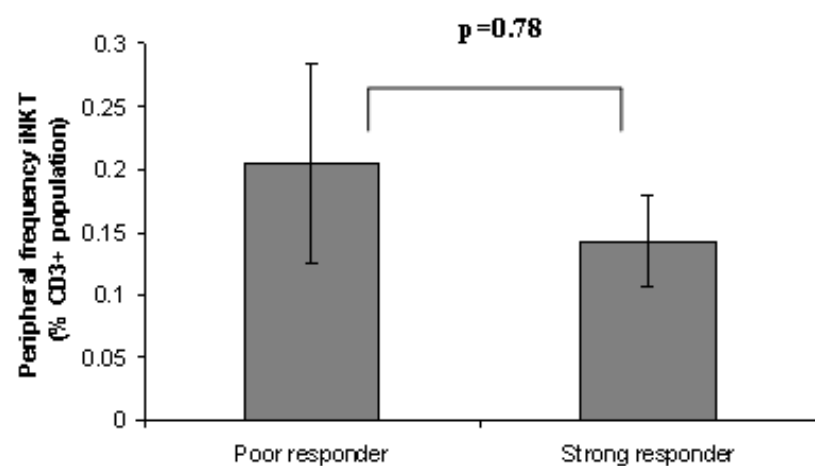

B

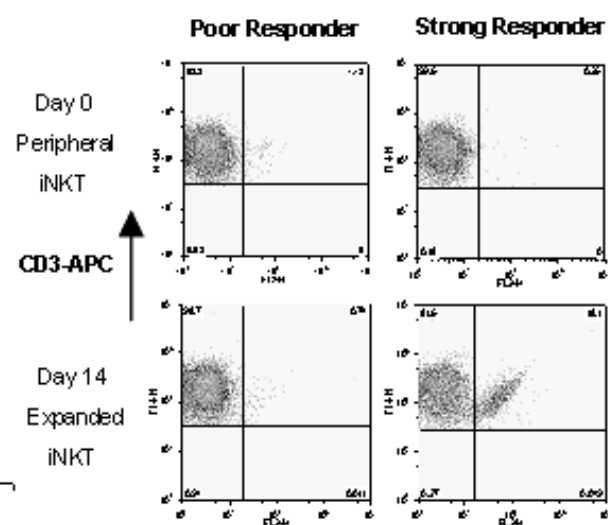

C

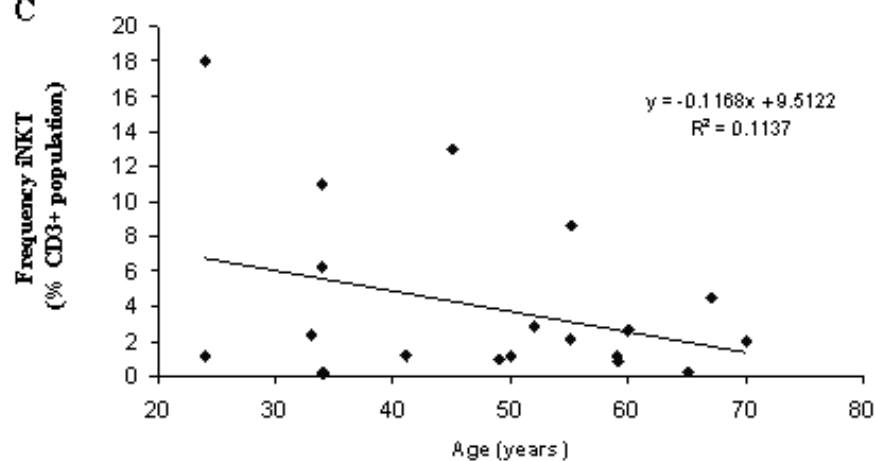

D

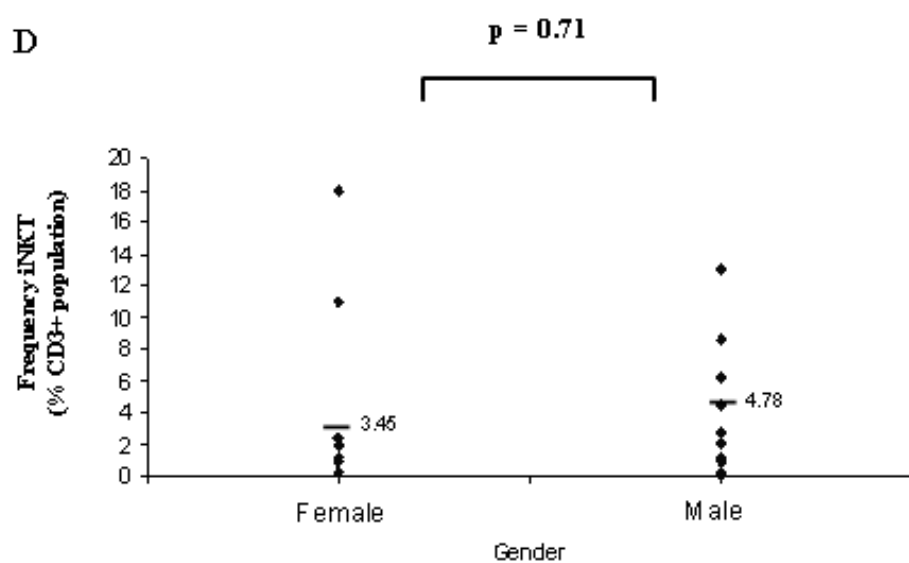

Supplement: Additional file 3 — Donor iNKT response phenotypes are not associated with peripheral iNKT levels, donor age or gender. (A) Comparison of peripheral iNKT cell levels between 'strong' and 'poor' iNKT responsive donor groups (n = 15 'poor' and n = 10 for 'strong' +/- standard error of the mean), (B) FACS plots illustrating relatively low peripheral levels in a 'strong' responder donor and relatively high peripheral levels in a 'poor' responder donor, (C) Comparison of donor age with iNKT cell expansion efficiency (i.e. % iNKT in total CD3+ population following 14 days of culture with αGalCer + IL-2), (n = 20) (D) Percentage of iNKT cells induced in 14-day PBMC cultures of 11 male and 8 female donors in response to αGalCer/IL-2. [file 1471-2172-9-71-S3.pdf]

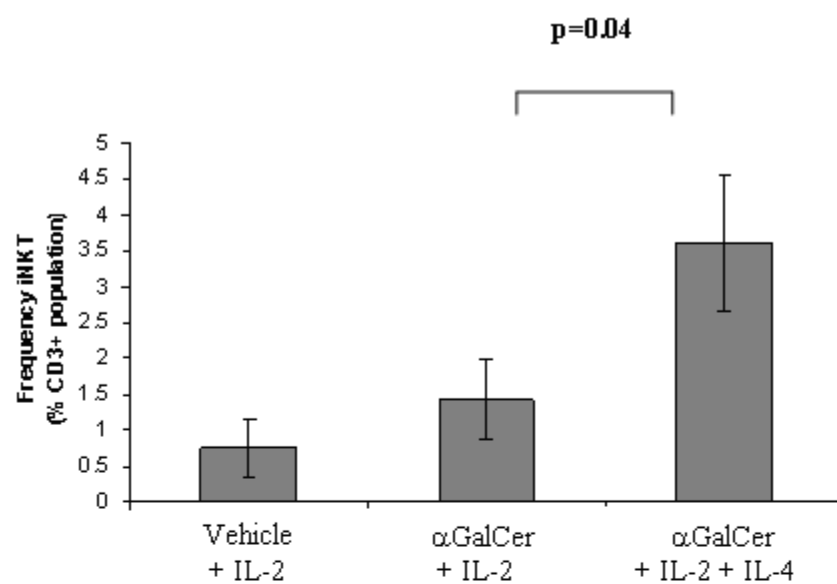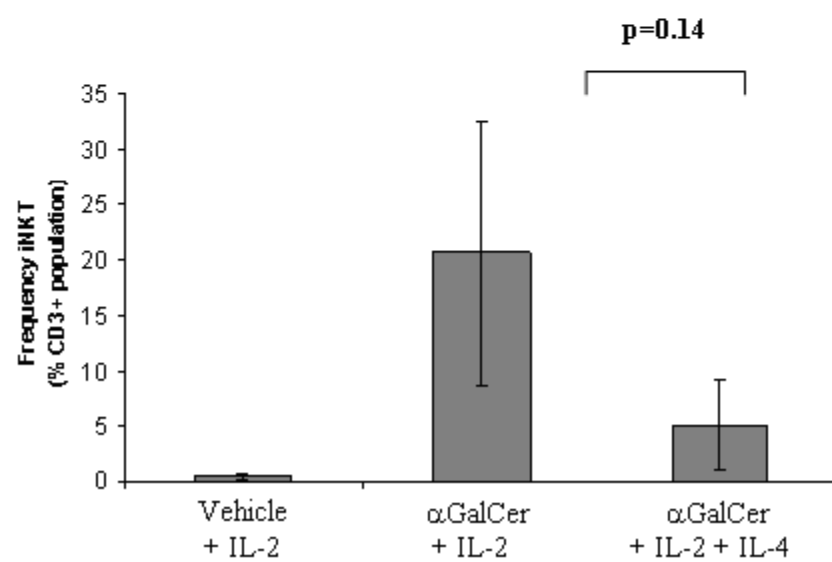

Supplement: Additional file 4 — Effects of exogenous IL-4 on expansion of iNKT cells in 'poor' and 'strong' responder donors. (A) Levels of PBMC-derived iNKT cells from 'poor' responder donors after 14-days of culture in the presence of: (i) vehicle (DMSO) + IL-2, (ii) αGalCer + IL-2, or (iii) αGalCer + IL-2 + IL-4 (n = 4, +/- standard error of the mean), (B) Levels of PBMC-derived iNKT cells from 'strong' responder donors after 14-days of culture in the presence of: (i) vehicle (DMSO) + IL-2, (ii) αGalCer + IL-2, or (iii) αGalCer + IL-2 + IL-4 (n = 3, +/- standard error of the mean). [file 1471-2172-9-71-S4.pdf]
